# Review of the implementation of plasma ctDNA testing on behalf of IQN Path ASBL: a perspective from an EQA providers’ survey

**DOI:** 10.1007/s00428-017-2222-z

**Published:** 2017-08-25

**Authors:** Zandra C. Deans, Hannah Williams, Elisabeth M. C. Dequeker, Cleo Keppens, Nicola Normanno, Ed Schuuring, Simon J. Patton, Melanie Cheetham, Rachel Butler, Jacqueline A. Hall, On behalf of IQN Path ASBL

**Affiliations:** 1 UK NEQAS for Molecular Genetics, Department of Laboratory Medicine, Royal Infirmary of Edinburgh, Little France Crescent, Edinburgh, EH16 4SA UK; 2 Biomedical Quality Assurance Research Unit, Department of Public Health and Primary Care, KU Leuven / University Hospital of Leuven, Leuven, Belgium; 3 Biomedical Quality Assurance Research Unit, Department of Public Health and Primary Care, KU Leuven, Leuven, Belgium; 4 Cell Biology and Biotherapy Unit, Instituto Nazionale Tumouri “Fondazione Giovanni Pascale”, IRCCS, Naples, Italy; 5 Department of Pathology, University of Groningen, University Medical Center of Groningen, Groningen, The Netherlands; 6 European Molecular Genetics Quality Network, Manchester Centre for Genomic Medicine, St Mary’s Hospital, Manchester, M13 9WL UK; 7 All Wales Genetic Laboratory, Institute of Medical Genetics, University Hospital of Wales, Heath Park, Cardiff, CF14 4XW UK; 8 International Quality Network for Pathology (IQN Path ASBL), 3A Sentier de l’Esperance, L-1474 Luxembourg, Luxembourg; 9 Division of Cancer, Department of Surgery and Cancer, Imperial College London, London, UK

## Introduction

Molecular biomarker analysis for the personalised treatment of non-small cell lung cancer (NSCLC) and colorectal cancer (CRC) is becoming more common, due to the number and availability of molecular targets for predictive biomarker testing increasing [1]. Clinical laboratories must implement accurate test procedures and provide timely and reliable test results, to ensure that appropriate therapies are administered to patients [2]. The challenge for laboratories is to keep pace with molecular biomarker developments while maintaining excellence in service standards.

Plasma circulating tumour DNA (ctDNA) may be found in the blood of cancer patients, alongside a larger fraction of circulating free DNA (cfDNA). Plasma ctDNA testing is becoming more common in the management of cancer patients [3]. It has several advantages: in the absence of suitable or sufficient tissue biopsy, it yields material for molecular analysis, can demonstrate molecular resistance to targeted treatment and is an alternative to invasive tissue sampling [4]*.* Plasma ctDNA analysis may also prove useful in cases of intra- and inter-tumour heterogeneity [5]*.* With formal approval from the European Medicine Agency (EMA), several clinical applications for plasma ctDNA testing are now being considered, including the detection of Epidermal Growth Factor Receptor (*EGFR)* mutations in the plasma of patients with advanced NSCLC [6]*.*

The implementation of new methods such as plasma ctDNA testing can be challenging for diagnostic laboratories. Indeed, it has been shown that inexperience in specialised and complex techniques can compromise the result quality [2, 7]. To address these issues, four EQA providers came together under the umbrella organisation the International Quality Network for Pathology IQN Path (IQN Path): Association Italiana di Oncologia Medica (AIOM), European Molecular Genetics Quality Network (EMQN), European Society of Pathology (ESP) EQA and the United Kingdom National External Quality Assessment Service (UK NEQAS) for Molecular Genetics. Their aim was to survey testing methods currently in use and to pilot an EQA which assessed the standards of plasma ctDNA testing. This article summarises the results of the survey, which evaluated current laboratory practices in this field and which will subsequently inform the design of a pilot EQA scheme for plasma ctDNA testing.

## Methods

An online survey of plasma ctDNA testing practice was designed by the IQN Path collaborative group. The survey was circulated by the four EQA members to their global network of participants, EMQN (1480), UK NEQAS (500), AIOM (47) and ESP testing schemes (568). The survey comprised six sections which included questions about laboratory participation in EQA for solid tumour testing in NSCLC and CRC, current experience and technologies used in plasma ctDNA testing and any analytical limitations of current test methodologies. The survey opened for completion between February 2016 and the middle of March 2016. The responses were analysed to understand current practices in the field of *EGFR* and *RAS* mutation testing using ctDNA and will inform design of future pilot EQA scheme.

## Results

Completed surveys were received from 167 laboratories. The submitted data was collated and summarised.

**Fig. 1:**
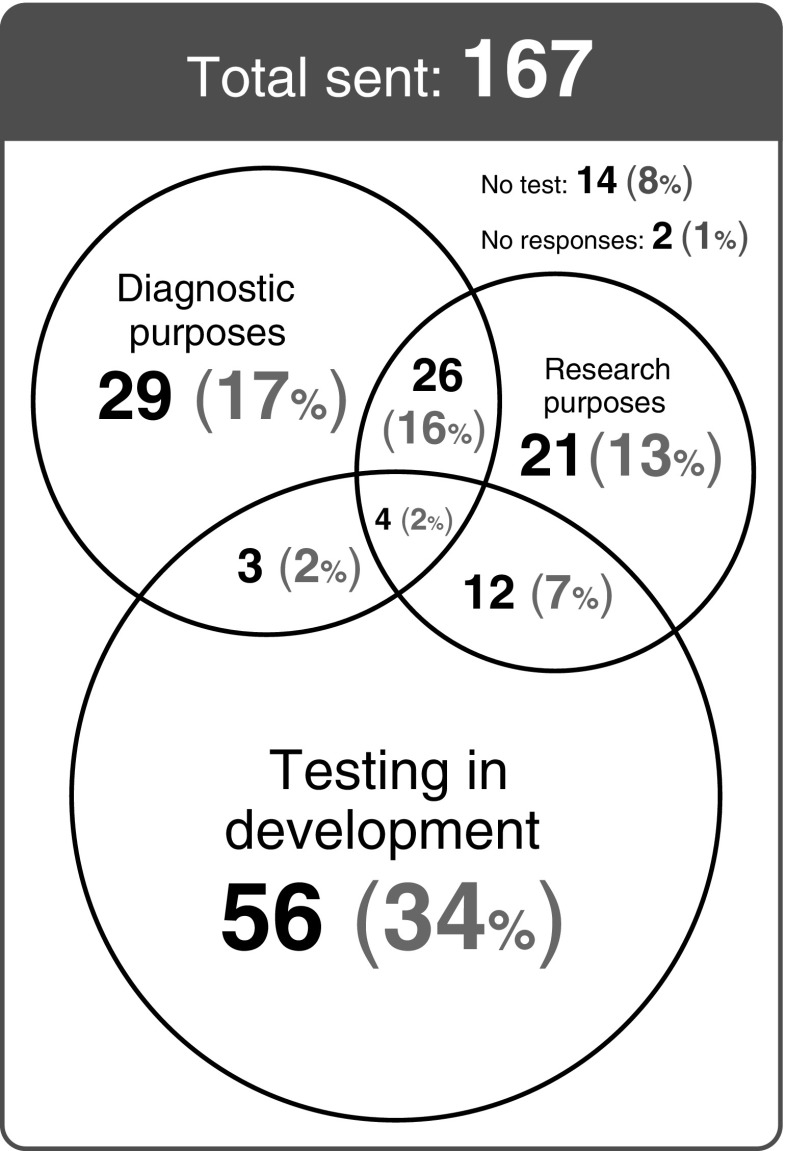
Summary of the survey results of *EGFR*, *KRAS and NRAS* mutation testing in plasma samples. Figures reported are based on the number of laboratories offering testing for either research or diagnostic use or have testing in the development phase. Note: some laboratories use testing for more than one purpose as may be seen by the intersections in the Venn diagram

The survey showed that some form of ctDNA plasma testing for *EGFR*, *KRAS* and *NRAS* was used in the majority of responding laboratories (151/167, 90%) but that only 62 (37%) laboratories currently perform diagnostic plasma ctDNA testing (Fig. 1). A further 56 laboratories (34%) have plasma ctDNA test methodologies in the development phase (Fig. 1). During 2015, 46 diagnostic laboratories tested fewer than 100 samples, while 9 tested more than 101 samples (4 did not respond).

**Fig. 2:**
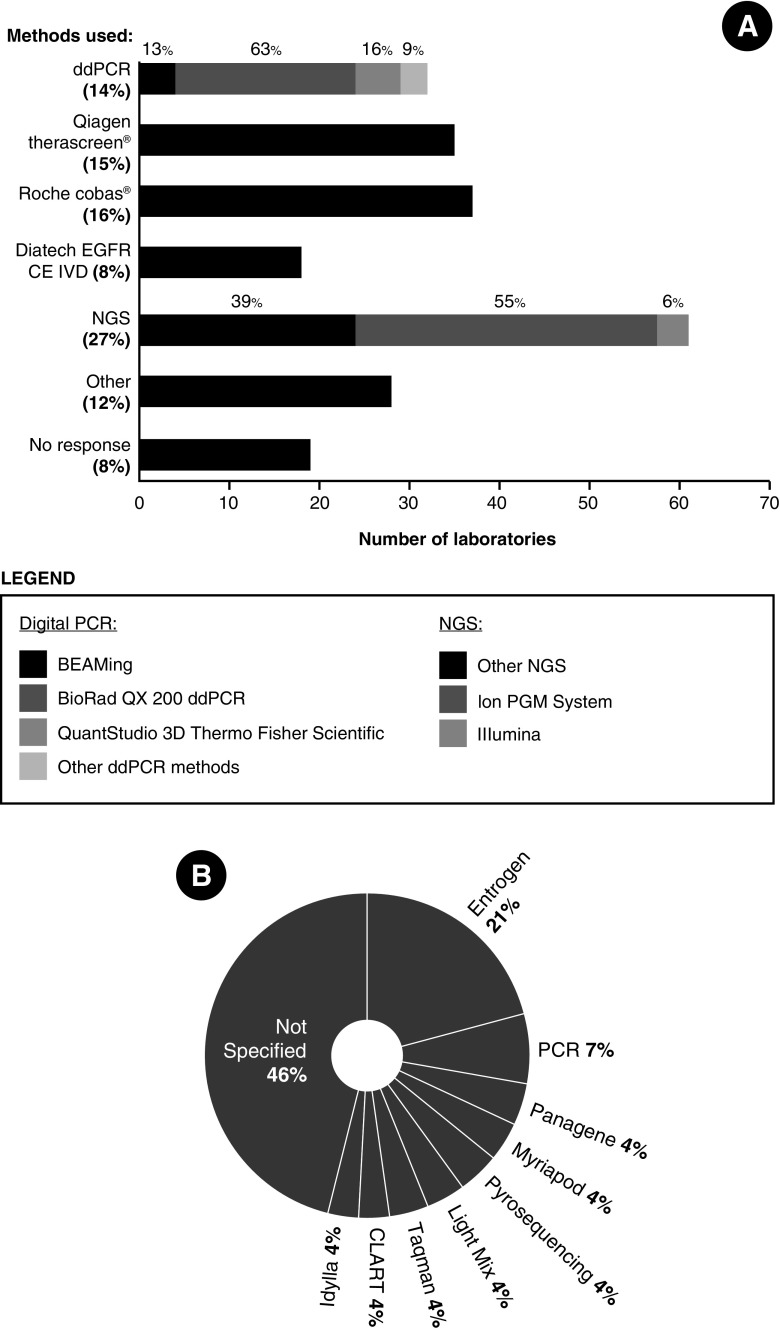
The breakdown of methodologies used for mutation testing in plasma samples. Values represent the number of laboratories running a specific platform. Some laboratories use more than one testing method. **a** The current methods used for plasma ctDNA mutation testing by number of laboratories (% included). **b** A detailed breakdown of methods specified as “other testing methods”

The most frequently used method for plasma ctDNA testing was next-generation sequencing (NGS), used by 27% of laboratories. The most commonly used testing platform was Ion PGM System®/Thermo Fisher Scientific (Fig. 2a). After NGS, the most frequently used methods were Roche cobas®, Qiagen therascreen® and ddPCR (Fig. 2a). Of the ddPCR assays, BioRad’s QX 200 ddPCR assays were the most commonly used (Fig. 2a).

Of the laboratories using more than one plasma ctDNA testing method, 90 (54%) employ a single method, 51 (31%) use two and 8 (5%) use three different methods. The remaining 18 did not provide methodology information. Figure 2b illustrates the diversity of methods currently employed in plasma ctDNA testing.

The stated limit of detection (LoD) of all allele frequencies for all laboratories was below 20%. The LoD was 5–20% in 13 laboratories (7.8%), < 5% in 56 laboratories (33.5%) and < 1% in 62 laboratories (37.1%). The remaining laboratories did not provide any LoD data (21.6%). Most laboratories performing NGS (97.8%) provided an LoD level of < 5% (44.2%), < 1% (53.8%) and 1% (< 5%). The other laboratories stated an LoD level of > 20% or provided no data. For ddPCR, 65.6% of laboratories gave an LoD of < 1 and 21.8% stated an LoD between 1 and < 5%. A single laboratory stated an LoD of < 10% and the remaining 3 offered no data.

The *EGFR*, *KRAS* and *NRAS* mutations targeted for analysis in plasma samples were collected. The three most common targets for each gene are outlined in Table 1.

**Table 1:** Tabulated frequency of the genes and variants analysed by laboratories for ctDNA testing

Gene	Target	Number of laboratory responses
*EGFR*	All variants within specified exons	61
	p.(G719A), p.(G719C), p.(G719S), p.(S768I), p.(T790M), p.(L858R), p.(L861Q), deletions in exon 19 and insertions in exon 20	28
	p.(T790M), p.(L858R) and deletions in exon 19	27
*KRAS*	All variants within specified exons	62
	p.(G12D), p.(G12R), p.(G12A), p.(G12C), p.(G12S), p.(G12V), p.(G13D) and p.(Q61H)	13
	p.(G12D), p.(G12R), p.(G12A), p.(G12C), p.(G12S), p.(G12V) and p.(G13D)	11
*NRAS*	All variants within specified exons	57
	p.(G12D), p.(Q61K), p.(Q61R), p.(Q61L) and p.(Q61H)	10
	All variants in codons 12, 13, 59, 117 and 146	4

## Discussion

Current practice for plasma ctDNA testing in CRC and NSCLC tumour diagnostic testing was examined by the survey. The laboratories offering plasma ctDNA testing were those which already provide tissue-based molecular pathology services. Most survey participants (86.8%) offered diagnostic testing of *EGFR*, *KRAS* and *NRAS* from formalin-fixed paraffin-embedded (FFPE) tissue and participated in EQA for solid tumours. Of these, 78% participated in EQA assessment for NSCLC and 68% for CRC, so these laboratories already have experience in molecular technologies and understand how to interpret and report results.

The data suggest that no single, definitive technology for the analysis of plasma ctDNA has yet emerged. The methods currently used are a mixture of commercial and locally developed assays. These assays must be optimised and validated: they must also support adequate test sensitivity and available starting material and must also cover the range of molecular targets that require analysis.

Plasma ctDNA is present at low quantities, mixed within circulating free DNA (cfDNA) in the blood. Therefore, in order to have confidence in the results of plasma ctDNA testing, attention must be paid to the assay sensitivity. However, the optimal sensitivity for ctDNA testing is not yet clear.

An example of a specific clinical application of plasma ctDNA is the phase IV *EGFR* tyrosine kinase inhibitor Gefitinib ‘Follow Up Measure’ trial that facilitated the approval of plasma ctDNA testing for *EGFR* in NSCLC patients (6). The trial showed that although the Qiagen therascreen® kit had a low sensitivity (65.7%), it had a good correlation with the response of patients to first-line treatment with Gefitinib [4].

In the future, more sensitive techniques are likely to detect more patients with *EGFR* mutations and may also identify *EGFR* variants in patients with heterogeneous expression [8].

The use of highly sensitive ctDNA testing methods has permitted new insights into heterogeneity, e.g. *p.T790M* mutations in the ctDNA of patients with a tumour mass that tested negative for the resistance mutation [9]. Patients with positive plasma ctDNA tests and negative tissue results had shorter progression-free survival compared to patients with *EGFR p.T790M* detected in both their tumour tissue and plasma ctDNA [9]. In the future, accurate measurements of the ratio of resistant *EGFR* mutations to sensitising mutations might help select patients who are more likely to benefit from treatment with drugs targeting *p.T790M* [10].

Similarly, NGS panels may be used to determine the relative abundance of a tumour variant to support individually tailored therapy. Where NGS is used, a broad range of molecular targets may be detected simultaneously; however, this may be at the cost of lower test LoD. Non-NGS-based methods may provide greater sensitivity but have the limitation of assaying fewer molecular targets.

There is a significant interest in the development of plasma ctDNA services. However, despite the 2014 EMA approval for plasma ctDNA biopsies which determine the suitability of first-line treatment of NSCLC with Gefitinib, few laboratories currently deliver NSCLC or CRC clinical diagnostic services [11]. For plasma ctDNA testing to become integrated into routine practice, those offering clinical services must be educated on its applications. However, until local services can validate and embed testing in patient pathways, laboratory and clinical uptake of plasma ctDNA may be hindered.

Current clinical applications for ctDNA are largely confined to NSCLC and CRC, although there is potential for its use in many other areas of oncology. Laboratories must provide high-quality testing services in which clinical teams and patients have confidence. The delivery of National and International EQA schemes is essential to maintain quality through the standardisation of sample logistics, molecular assays and result interpretation, as well as playing an important role in supporting education [4].

As many laboratories plan to implement the testing of plasma ctDNA, it is clear that support from well-designed EQA schemes is needed. Without this support, laboratories may be slower to offer plasma ctDNA clinical services or may encounter issues. Surveying current practices and collecting data to inform EQA design is a task that may be harmonised between several EQA providers, all with the aim of increasing efficiencies and supporting best practice standards in quality assessment [12].

## Electronic supplementary material

ESM 1 (DOCX 81 kb)
