# Supplementary material for: Review of the implementation of plasma ctDNA testing on behalf of IQN Path ASBL: a perspective from an EQA providers’ survey
Source: Virchows Arch. 2017 Aug 25;471(6):809–13. doi: 10.1007/s00428-017-2222-z (PMC5711978; doi:10.1007/s00428-017-2222-z)
Supplement: Supplementary file 1 — (DOCX 81 kb) [file 428_2017_2222_MOESM1_ESM.docx]

**ctDNA Survey: Supplementary data**

| **No.** | **Section** | **Questions** |
| --- | --- | --- |
| 1 | Contact Information | Full laboratory contact details |
|  |  | Confirmation of EQA provider source of invitation email for survey |
| 2 | Laboratory experience | Accreditation status of laboratory (accredited/not accredited/in process of accreditation) |
|  |  | Accreditation body (if relevant) |
|  |  | Accreditation standards (if relevant) |
| 3 | Participation in EQA | Participation, or not, in an EQA scheme for solid tumour testing in NSCLC |
|  |  | Participation, or not, in an EQA scheme for solid tumour testing in CRC |
| 4 | Experience of ctDNA testing for *EGFR* and RAS | Currently offering diagnostic testing for *EGFR* and *RAS* from tissue FFPE |
|  |  | Currently offering testing for *EGFR* and *RAS* from plasma / serum and context (diagnostic/ research/in development) |
|  |  | Number of ctDNA tests for *EGFR* and RAS undertaken between Jan-Dec 2015 in each context (diagnostic/ research/in development) |
| 5 | Technologies used for plasma analysis | A selection from ddPCR, Qiagen therascreen^®^, Roche cobas^®^, NGS and other technologies |
|  |  | Establish manufacturers of NGS and ddPCR platforms |
| 6 | Mutations detected and analytical sensitivity | Establish which *EGFR* mutations assay tests for in plasma |
|  |  | Establish which *KRAS* mutations assay tests for in plasma |
|  |  | Establish which *NRAS* mutations assay tests for in plasma |
|  |  | Establish assay sensitivity and limit of detection (LoD) |
